# Association between the neutrophil-to-lymphocyte ratio and the incidence of diabetic retinopathy: a systematic review and meta-analysis

**DOI:** 10.3389/fendo.2025.1712767

**Published:** 2026-01-12

**Authors:** Wenshu Jin, Xiaofang Tu, Xiaopei Chen, Jinghong Zhang

**Affiliations:** Department of Endocrinology, Zhejiang Hospital, Hangzhou, Zhejiang, China

**Keywords:** diabetes mellitus, diabetic retinopathy, meta-analysis, neutrophil-to-lymphocyte ratio, systematic review

## Abstract

**Background:**

Accumulating evidence has indicated a possible relation of the neutrophil-to-lymphocyte ratio (NLR) to diabetic retinopathy (DR) incidence. However, current findings remain inconclusive.

**Methods:**

PubMed, Embase, Web of Science, as well as Cochrane Library, were thoroughly retrieved before March 20, 2025, for eligible studies examining the relation of NLR to DR incidence. The primary outcomes included DR incidence assessed as a categorical and a continuous variable. Categorical data were expressed as odds ratios (ORs) with 95% confidence intervals (CIs), and continuous data were expressed as standardized mean differences (SMDs) with 95% CIs.

**Results:**

35 studies comprising 49,664 patients were encompassed. Pooled results demonstrated a significant relation between elevated NLR level and a greater DR risk, when analyzed as a categorical variable (OR = 1.48, 95% CI: 1.34-1.64; *p* < 0.00001) and as a continuous variable (SMD = 0.47, 95% CI: 0.36-0.59; *p* < 0.00001). Sensitivity analyses demonstrated the robustness of our findings. Subgroup analyses showed that regional differences were the main source of heterogeneity.

**Conclusions:**

An increased NLR may be significantly related to a greater risk of DR among individuals with diabetes. However, further prospective and multicenter investigations are necessary to corroborate these findings.

**Systematic Review Registration:**

https://www.crd.york.ac.uk/prospero/display_record.php?RecordID=1054742, identifier CRD420251054742.

## 1 Introduction

Diabetes mellitus (DM) is a slowly progressive systemic metabolic disorder, and long-term hyperglycemia can lead to various complications (1). Diabetic retinopathy (DR), among the most common microvascular complications of DM, is a predominant cause of blindness and visual impairment (2, 3) among working-age people worldwide. A meta-analysis encompassing 59 studies across 27 countries has reported that DR affects approximately 22.27% of individuals with diabetes worldwide, with an estimated 103.12 million DR adults in 2020. This figure is forecast to reach 160.5 million by 2045 (4). Timely DR screening is crucial to prevent irreversible vision loss, but DR patients frequently remain asymptomatic during the initial stages, and DR screening equipment is expensive and difficult to popularize (5). The pathophysiological mechanisms underlying DR are not yet fully elucidated and entail blood-retinal barrier disruption, retinal neurodegeneration, oxidative stress (OS), chronic low-grade inflammation, genetic predisposition, and immune-mediated processes, among other factors (6, 7). Epidemiological studies have identified several risk factors significantly related to DR development, like prolonged duration of diabetes, poor glycemic control, hypertension, nephropathy, dyslipidemia, smoking, and rising body mass index (BMI) (8). Nonetheless, these factors alone do not comprehensively explain the complex pathogenesis of DR.

Chronic low-grade inflammation is crucial in DR development (9). More inflammatory cytokines and chemokines have been detected in various biological specimens, like serum, vitreous fluid, aqueous humor, and retinal tissue, from individuals with DR (10). Subpopulations of leukocytes are increasingly recognized as biomarkers of systemic inflammation (11). Among them, the neutrophil-to-lymphocyte ratio (NLR) has garnered attention due to its simplicity and ease of measurement (12). Increased NLR is linked to a wide variety of pathological conditions, including infectious diseases, cardiovascular disorders, autoimmune diseases, malignancies, and systemic inflammation (13). Moreover, elevated NLR levels have been linked to diabetic nephropathy (DN) (14). Given the shared pathophysiological pathways and frequent co-occurrence of DR and DN (15), it is hypothesized that NLR correlated with DR. Tang et al.’s 2018–2021 cohort study (16) demonstrated a positive correlation between baseline NLR levels and the risk of DR in the type 2 diabetes mellitus (T2DM) population, with every 1-standard deviation (SD) rise in NLR corresponding to a 29.2% elevated risk of DR. Consistently, Deng et al. (17) reported higher NLR values among T2DM patients in the non-proliferative DR (NPDR) and proliferative DR (PDR) cohorts than those without DR. In contrast, a separate study involving 4,813 T2DM patients proved the relation of NLR to DN but not to DR (18), thereby highlighting the ongoing controversy regarding the link of NLR to DR.

Luo et al. (19) and Harley et al. (20) conducted meta-analyses on blood cell-related inflammatory markers and their association with DR. Regarding the association between NLR and the incidence of DR, Luo et al. synthesized 10 studies published before 2017, whereas Harley et al. incorporated 16 studies up to 2024. Since the publication of these meta-analyses, a growing body of clinical research has emerged to further examine the NLR–DR relationship; however, the findings of these more recent investigations have not been fully consistent with earlier conclusions. Accordingly, the present study aims to reassess whether NLR serves as a reliable predictor of DR incidence by integrating evidence from the latest and most comprehensive clinical studies on the basis of previous meta-analytic work, and to evaluate the robustness and credibility of the evidence through sensitivity analyses and stratified subgroup analyses.

## 2 Methods

### 2.1 Literature search

Our study followed the Preferred Reporting Items for Systematic Reviews and Meta-Analyses (PRISMA 2020) guidelines (21), with the protocol registered on the International Prospective Systematic Evaluation Registry (PROSPERO: CRD420251054742). Two investigators, JWS and TXF, devised the search strategy. They independently utilized subject terms and keywords including “diabetic retinopathy,” “diabetic retinopathies,” “neutrophil(s),” “polymorphonuclear neutrophil(s),” “polymorphonuclear leukocyte(s),” “LE cells,” “LE cell,” “neutrophil band cell(s),” “lymphocyte(s),” “lymphoid cell(s),” “ratio”, for search across PubMed, Embase, Web of Science, and Cochrane Library until March 20, 2025. The PubMed retrieval strategy was as follows: ((((“Neutrophils”[Mesh]) OR (Neutrophil OR Polymorphonuclear Neutrophils OR Polymorphonuclear Neutrophil OR Polymorphonuclear Leukocyte OR Polymorphonuclear Leukocytes OR LE Cells OR LE Cell OR Neutrophil Band Cells OR Neutrophil Band Cell)) AND ((“Lymphocytes”[Mesh]) OR (Lymphocyte OR Lymphoid Cells OR Lymphoid Cell))) AND Ratio) AND ((Diabetic Retinopathies) OR “Diabetic Retinopathy”[Mesh]). The strategy is provided in **Supplementary Table S1**.

### 2.2 Study selection

Inclusion criteria were: (1) P (Population): patients with DM (with or without DR); (2) E (Exposure): high NLR in cohort studies and DR status in case-control studies; (3) C (Comparison): low NLR for cohort studies and non-DR status for case-control studies; (4) O (Outcome): incidence rate of DR with data on odd ratio (OR) with a 95% confidence interval (CI) or mean ± SD, which could be extracted or computed; (5) S (Study design): cohort or case-control studies.

Exclusion criteria were: (1) Reviews, comments, conference abstracts, case reports, as well as letters; (2) No data to derive OR and 95% CI or mean ± SD; (3) Duplicate or overlapping information; (4) Non-English studies.

JWS and TXF independently checked titles and abstracts, downloaded relevant full texts, and appraised them for inclusion. Dissents were addressed via discussion.

### 2.3 Data extraction

Data were extracted by JWS and TXF independently. Dissents were settled via consensus. Extracted information encompassed first author, publication year, duration, country (location), design, sample size, age, sex, BMI, glycated hemoglobin (HbA1c), fasting blood glucose (FBG), NLR cut-off value, ORs (95% CIs), and mean ± SD for DR incidence.

### 2.4 Quality assessment

Study quality was rated via the Newcastle-Ottawa Quality Assessment Scale (NOS) (22) in selection, comparability, and outcomes (Exposure), with the maximum score being nine for each study. 7–9 suggested high quality, 4–6 suggested moderate quality, and 1–3 suggested low quality. For low-quality studies, subgroup analyses based on study quality were conducted. Two investigators (JWS and TXF) independently assessed the quality of the included studies, with results reviewed by a third investigator (ZJH). Disagreement was resolved through discussion.

### 2.5 Statistical analysis

The link of NLR to DR incidence among the DM population was explored via ORs for DR (categorical variables) and standardized mean differences (SMDs) for DR (continuous variables), with 95% CIs. Heterogeneity was assessed utilizing Cochran’s Q test and Higgins’ *I^2^* statistic (23). *I^2^* > 50% or *p* < 0.1 denotes significant heterogeneity. Every data analysis was enabled via a random-effects model. We conducted sensitivity analyses by sequentially excluding individual studies to assess the robustness of our findings. Subgroup analyses were conducted by examining study design, age, region, and NLR cut-off values to investigate potential sources of heterogeneity. Publication bias was detected utilizing funnel plots and Egger’s tests. *p* < 0.05 signified statistical significance. Our statistical analyses were enabled by STATA 15.0 and Review Manager 5.4.

## 3 Results

### 3.1 Study characteristics

**Figure 1:**
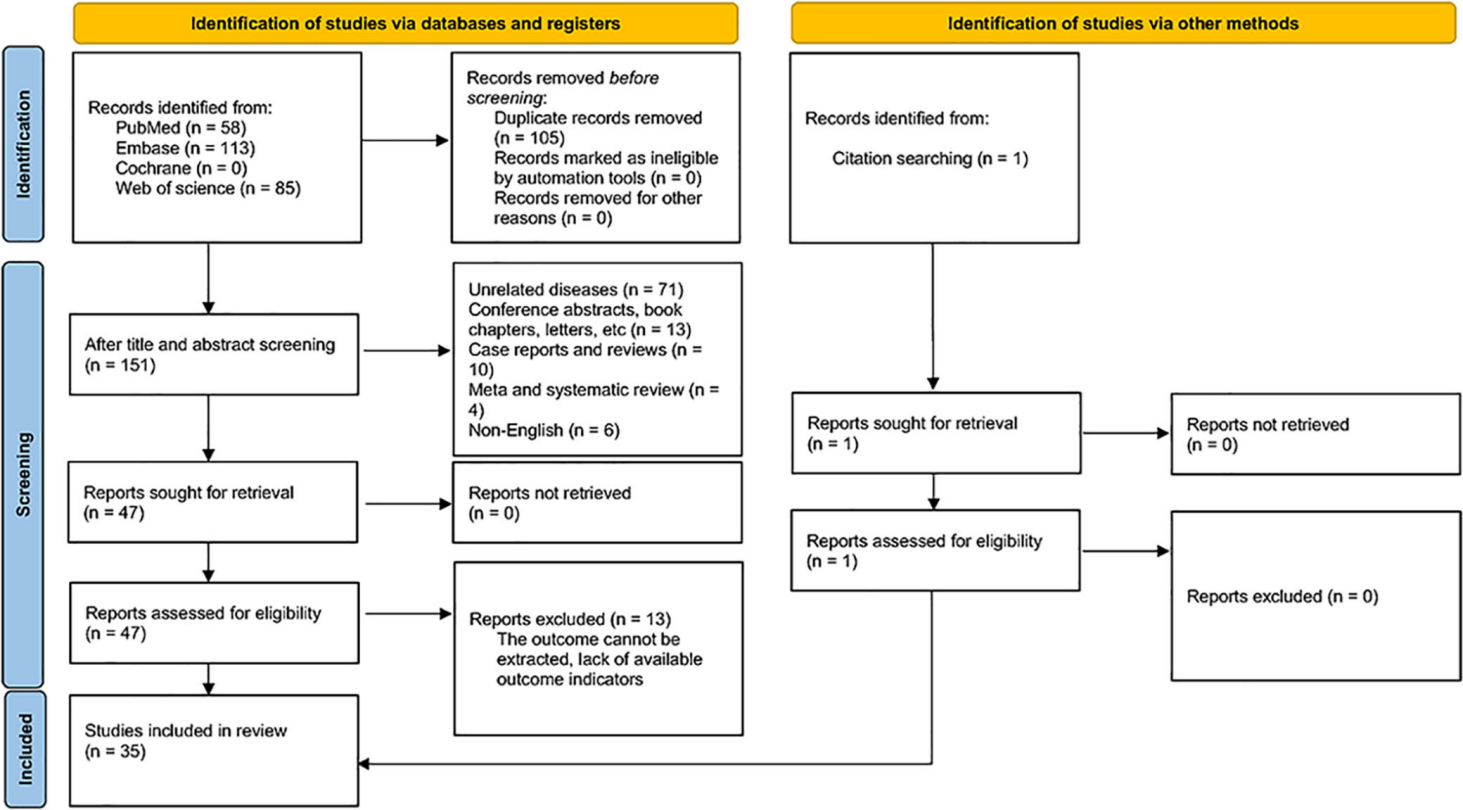
Flow chart of literature screening.

256 articles were initially retrieved. 105 were subsequently removed for duplicates, and 104 were ostracized upon title and abstract review of the rest. The full texts of 47 studies were subsequently checked, with 13 excluded due to inadequate data. Additionally, one article was identified through citation searching. Ultimately, 35 studies were eligible (11, 16–18, 24–54), involving 49,664 patients in total (**Figure 1**).

74 comparison groups were extracted from 35 included studies. One study was conducted in America, one in Japan, one in Scotland, one in Iraq, one in Iran, two in Romania, three in India, nine in Turkey, and the remaining sixteen studies were conducted in China. Among these, two were cohort studies (16, 44), whereas the remainder were case-control studies (11, 17, 18, 24–43, 45–54). All studies, including cohort studies, employed retrospective designs. Notably, 13 eligible articles (11, 17, 18, 24, 25, 29, 32, 34, 37, 38, 51–53) encompassed thirty-four comparison groups stratified by DR subtypes like NPDR, PDR, DR with diabetic macular edema (DME), and DR without DME. In addition, six articles (16, 18, 30, 35, 39, 48) encompassed twenty-three comparison groups categorized by different NLR cut-off values, yielding a total of seventy-four comparison groups. The mean age of participants ranged from 48.13 to 68.33. Their mean BMI was 24.00-32.44 kg/m², and their mean HbA1c was 7.10%-9.84%. The duration of diabetes ranged from 3 to 16.19 years. All were English publications between 2013 and 2025. The cohort studies assessed DR incidence by comparing groups with high versus low NLR levels, whereas the case-control studies evaluated NLR in diabetic patients with and without DR. The characteristics are presented in **Table 1**.

**Table 1:** Basic characteristics of the included literature.

First author and year	Duration	Country	Study design	Sample size	Gender		Age	BMI (kg/m^2^)	HbA1C (%)	FBG (mg/dL)	NLR cut-off	Variables adjusted
					Male	Female						
Abshenas 2022a (24)	14.17	Iran	Case-control study	103	42	61	59.73	NA	8.89	NA	NA	NA
Abshenas 2022b (24)	14.17	Iran	Case-control study	103	42	61	59.73	NA	8.89	NA	NA	NA
Atli 2022a (25)	11.09	Turkey	Case-control study	60	25	35	58.66	28.97	9.50	172.17	NA	NA
Atli 2022b (25)	11.09	Turkey	Case-control study	60	25	35	58.66	28.97	9.50	172.17	NA	NA
Bhattacharyya 2021 (26)	8.62	India	Case-control study	80	47	33	59.89	NA	7.10	NA	3.53	NA
Çakir 2020 (27)	NA	Turkey	Case-control study	122	0	122	60.53	NA	8.40	NA	NA	NA
Chen 2024 (28)	11.30	China	Case-control study	719	719	0	64.55	25.44	8.09	179.80	NA	age, smoking, diabetic kidney disease, diabetic neuropathy, fasting blood glucose, glycated albumin, hsCRP
Chen 2023a (29)	7.44	China	Case-control study	500	268	232	56.00	26.01	8.85	151.92	NA	NA
Chen 2023b (29)	7.44	China	Case-control study	500	268	232	56.00	26.01	8.85	151.92	NA	NA
Chen 2023c (29)	7.44	China	Case-control study	500	268	232	56.00	26.01	8.85	151.92	NA	NA
Chittawar 2017a (30)	3.00	India	Case-control study	265	121	144	51.12	25.93	8.55	187.25	1.71	NA
Chittawar 2017b (30)	3.00	India	Case-control study	265	121	144	51.12	25.93	8.55	187.25	2.10	NA
Chittawar 2017c (30)	3.00	India	Case-control study	265	121	144	51.12	25.93	8.55	187.25	2.60	NA
Ciray 2015 (31)	9.10	Turkey	Case-control study	114	41	73	59.70	30.30	9.18	NA	NA	NA
Dascalu 2023I-a (11)	NA	Romania	Case-control study	90	35	55	65.10	NA	7.20	163.23	NA	diabetic nephropathy, creatinine, PLR, HbA1c, neutrophils, thrombocytes
Dascalu 2023I-b (11)	NA	Romania	Case-control study	90	35	55	65.10	NA	7.20	163.23	NA	NA
Dascalu 2023II-a (32)	8.90	Romania	Case-control study	129	67	62	65.60	NA	7.60	164.60	3.18	duration
Dascalu 2023II-b (32)	8.90	Romania	Case-control study	129	67	62	65.60	NA	7.60	164.60	NA	NA
Deng 2025a (17)	NA	China	Case-control study	397	187	210	63.67	24.00	7.73	NA	2.56	age, albumin, creatinine, uric acid, urea, neutrophils, lymphocytes, SII, SIRI, AISI, PLR, MLR, sex
Deng 2025b (17)	NA	China	Case-control study	397	187	210	63.67	24.00	7.73	NA	2.56	age, albumin, creatinine, uric acid, urea, neutrophils, lymphocytes, SII, SIRI, AISI, PLR, MLR, sex
Erdem 2022 (33)	NA	Turkey	Case-control study	118	30	88	61.81	NA	NA	NA	NA	NA
Gao 2024a (34)	NA	China	Case-control study	141	78	63	52.82	NA	7.44	133.38	1.25	PLR, SII
Gao 2024b (34)	NA	China	Case-control study	141	78	63	52.82	NA	7.44	133.38	NA	NA
He 2022a (35)	10.74	United States	Case-control study	2772	1424	1348	61.30	32.44	7.51	NA	NA	age, gender, BMI, poverty income ratio, diabetes duration, marital status, stroke, coronary heart disease, heart failure
He 2022b (35)	10.74	United States	Case-control study	2772	1424	1348	61.30	32.44	7.51	NA	1.76	age, gender, BMI, poverty income ratio, diabetes duration, marital status, stroke, coronary heart disease, heart failure
He 2022c (35)	10.74	United States	Case-control study	2772	1424	1348	61.30	32.44	7.51	NA	2.57	age, gender, BMI, poverty income ratio, diabetes duration, marital status, stroke, coronary heart disease, heart failure
Huang 2022 (36)	10.00	China	Case-control study	195	110	85	54.77	NA	7.60	NA	NA	NA
Imga 2016a (37)	8.35	Turkey	Case-control study	91	29	62	51.55	29.78	8.05	NA	NA	NA
Imga 2016b (37)	8.35	Turkey	Case-control study	91	29	62	51.55	29.78	8.05	NA	NA	NA
Imga 2016c (37)	8.35	Turkey	Case-control study	91	29	62	51.55	29.78	8.05	NA	NA	NA
Imga 2016d (37)	8.35	Turkey	Case-control study	91	29	62	51.55	29.78	8.05	NA	NA	NA
Imga 2016e (37)	8.35	Turkey	Case-control study	91	29	62	51.55	29.78	8.05	NA	NA	NA
Kurtul 2022a (38)	9.09	Turkey	Case-control study	128	58	70	56.50	31.48	8.95	160.25	NA	age, duration, hypertension, creatinine, hemoglobin, prognostic nutritional index, PLR
Kurtul 2022b (38)	9.09	Turkey	Case-control study	128	58	70	56.50	31.48	8.95	160.25	NA	NA
Kurtul 2022c (38)	9.09	Turkey	Case-control study	128	58	70	56.50	31.48	8.95	160.25	NA	NA
Li 2024I-a (39)	9.32	China	Case-control study	1058	721	337	54.67	26.04	8.59	NA	2.33	age, body mass index, sex, diabetic duration,hypertension, dyslipidemia, and HbA1c
Li 2024I-b (39)	9.32	China	Case-control study	1058	721	337	54.67	26.04	8.59	NA	1.90	age, body mass index, sex, diabetic duration,hypertension, dyslipidemia, and HbA1c
Li 2024I-c (39)	9.32	China	Case-control study	1058	721	337	54.67	26.04	8.59	NA	2.66	age, body mass index, sex, diabetic duration,hypertension, dyslipidemia, and HbA1c
Li 2024II (40)	8.72	China	Case-control study	1182	473	709	63.16	27.06	7.38	NA	NA	age, systolic blood pressure, glucose, HbA1c, urea, creatinin, SIRI, duration, gender, stroke
Mahajan 2023 (41)	6.30	India	Case-control study	100	64	37	56.30	NA	8.90	NA	3.53	NA
Mineoka 2018 (42)	16.19	Japan	Case-control study	335	187	148	67.40	24.69	7.47	NA	NA	NA
Onalan 2019 (43)	NA	Turkey	Case-control study	100	48	52	56.34	NA	9.62	NA	NA	NA
Rajendrakumar 2023 (44)	NA	Scotland	Cohort study	23531	13013	10518	61.70	32.20	7.30	NA	3.04	sex, diastolic blood pressure, HbA1c, systolic blood pressure, age, non-high-density lipoprotein cholesterol, BMI, eGFR, diabetes drug
Sarhan 2023 (45)	NA	Iraq	Case-control study	160	66	94	55.52	30.89	NA	NA	NA	NA
Shan 2022 (46)	9.73	China	Case-control study	456	292	164	53.54	25.60	8.40	151.92	NA	age, diabetes duration, diabetic peripheral neuropathy, oral antihypertensive drugs, systolic blood pressure, total cholesterol, remnant cholesterol, microalbuminuria, triglyceride glucose index
Tang 2024a (16)	4.15	China	Cohort study	857	560	297	48.13	25.52	7.53	146.07	NA	age, sex, diabetes course, smoking history, drinking history, BMI, systolic blood pressure, triglyceride, HbA1c, fasting blood glucose, fasting insulin, uric acid, eGFR, UACR
Tang 2024b (16)	4.15	China	Cohort study	857	560	297	48.13	25.52	7.53	146.07	1.47	age, sex, diabetes course, smoking history, drinking history, BMI, systolic blood pressure, triglyceride, HbA1c, fasting blood glucose, fasting insulin, uric acid, eGFR, UACR
Tang 2024c (16)	4.15	China	Cohort study	857	560	297	48.13	25.52	7.53	146.07	1.91	age, sex, diabetes course, smoking history, drinking history, BMI, systolic blood pressure, triglyceride, HbA1c, fasting blood glucose, fasting insulin, uric acid, eGFR, UACR
Tang 2024d (16)	4.15	China	Cohort study	857	560	297	48.13	25.52	7.53	146.07	2.45	age, sex, diabetes course, smoking history, drinking history, BMI, systolic blood pressure, triglyceride, HbA1c, fasting blood glucose, fasting insulin, uric acid, eGFR, UACR
Ulu 2013 (47)	NA	Turkey	Case-control study	58	18	40	50.31	29.91	8.02	175.86	NA	NA
Wan 2020a (18)	8.50	China	Case-control study	4797	2212	2585	67.16	24.96	7.49	139.86	1.38	age, sex, education status, duration of diabetes, current smoking, BMI, HbA1c, dyslipidemia, and systolic blood pressure.
Wan 2020b (18)	8.50	China	Case-control study	4797	2212	2585	67.16	NA	7.49	139.86	1.38	age, sex, education status, duration of diabetes, current smoking, BMI, HbA1c, dyslipidemia, and systolic blood pressure
Wan 2020c (18)	8.50	China	Case-control study	4797	2212	2585	67.16	NA	7.49	139.86	1.38	age, sex, education status, duration of diabetes, current smoking, BMI, HbA1c, dyslipidemia, and systolic blood pressure
Wan 2020d (18)	8.50	China	Case-control study	4797	2212	2585	67.16	NA	7.49	139.86	1.77	age, sex, education status, duration of diabetes, current smoking, BMI, HbA1c, dyslipidemia, and systolic blood pressure
Wan 2020e (18)	8.50	China	Case-control study	4797	2212	2585	67.16	NA	7.49	139.86	1.77	age, sex, education status, duration of diabetes, current smoking, BMI, HbA1c, dyslipidemia, and systolic blood pressure
Wan 2020f (18)	8.50	China	Case-control study	4797	2212	2585	67.16	NA	7.49	139.86	1.77	age, sex, education status, duration of diabetes, current smoking, BMI, HbA1c, dyslipidemia, and systolic blood pressure
Wan 2020g (18)	8.50	China	Case-control study	4797	2212	2585	67.16	NA	7.49	139.86	2.30	age, sex, education status, duration of diabetes, current smoking, BMI, HbA1c, dyslipidemia, and systolic blood pressure
Wan 2020h (18)	8.50	China	Case-control study	4797	2212	2585	67.16	NA	7.49	139.86	2.30	age, sex, education status, duration of diabetes, current smoking, BMI, HbA1c, dyslipidemia, and systolic blood pressure
Wan 2020i (18)	8.50	China	Case-control study	4797	2212	2585	67.16	NA	7.49	139.86	2.30	age, sex, education status, duration of diabetes, current smoking, BMI, HbA1c, dyslipidemia, and systolic blood pressure
Wang 2015 (49)	NA	China	Case-control study	269	118	151	63.37	25.49	7.29	130.00	NA	NA
Wang 2020a (48)	8.17	China	Case-control study	470	277	193	56.02	24.34	9.84	166.67	NA	age, gender, diabetes duration, hypertension, BMI, serum creatinine, white blood cell count, hemoglobin, blood urea nitrogen, triglyceride, fasting plasma glucose and HbA1c
Wang 2020b (48)	8.17	China	Case-control study	470	277	193	56.02	24.34	9.84	166.67	1.50	age, gender, diabetes duration, hypertension, BMI, serum creatinine, white blood cell count, hemoglobin, blood urea nitrogen, triglyceride, fasting plasma glucose and HbA1c
Wang 2020c (48)	8.17	China	Case-control study	470	277	193	56.02	24.34	9.84	166.67	1.95	age, gender, diabetes duration, hypertension, BMI, serum creatinine, white blood cell count, hemoglobin, blood urea nitrogen, triglyceride, fasting plasma glucose and HbA1c
Wang 2020d (48)	8.17	China	Case-control study	470	277	193	56.02	24.34	9.84	166.67	2.54	age, gender, diabetes duration, hypertension, BMI, serum creatinine, white blood cell count, hemoglobin, blood urea nitrogen, triglyceride, fasting plasma glucose and HbA1c
Yang 2023 (50)	9.00	China	Case-control study	2610	1293	1317	63.00	24.20	8.90	172.98	NA	NA
Yeter 2022a (51)	6.50	Turkey	Case-control study	143	74	69	63.00	NA	8.44	NA	NA	NA
Yeter 2022b (51)	6.50	Turkey	Case-control study	143	74	69	63.00	NA	8.44	NA	NA	NA
Yue 2015a (52)	7.46	China	Case-control study	246	135	111	55.50	NA	7.50	154.80	NA	NA
Yue 2015b (52)	7.46	China	Case-control study	246	135	111	55.50	NA	7.50	154.80	NA	NA
Yue 2015c (52)	7.46	China	Case-control study	246	135	111	55.50	NA	7.50	154.80	NA	NA
Zeng 2022a (53)	6.08	China	Case-control study	290	143	147	56.33	24.07	9.15	NA	NA	NA
Zeng 2022b (53)	6.08	China	Case-control study	290	143	147	56.33	24.07	9.15	NA	NA	NA
Zeng 2022c (53)	6.08	China	Case-control study	290	143	147	56.33	24.07	9.15	NA	NA	NA
Zhang 2021 (54)	12.22	China	Case-control study	6978	2975	4003	68.33	25.02	7.25	133.38	NA	disease course, systolic blood pressure, HbA1c, blood glucose, mean platelet volume, hematocrit, mean corpuscular volume

### 3.2 Study quality

The encompassed studies had NOS scores of 6-9, indicating an overall moderate to high quality, with no low-quality studies.(**Supplementary Table S2**).

### 3.3 Meta-analysis results

#### 3.3.1 NLR and DR incidence (categorical variables)

**Figure 2:**
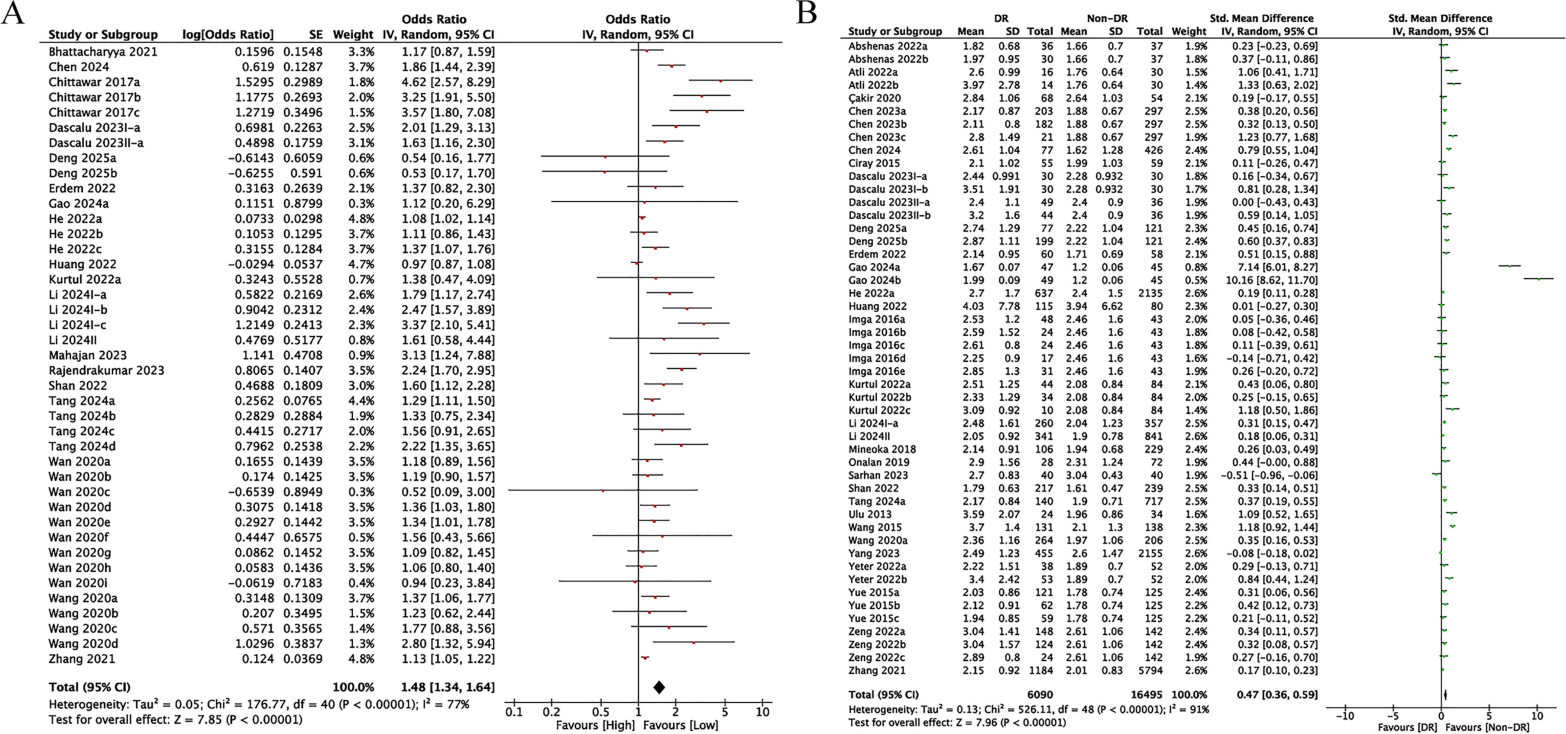
**(A)** Forest plots for the association between NLR and DR incidence (categorical variables); **(B)** Forest plots for the association between NLR and DR incidence (continuous variables).

Among the encompassed comparison groups, 41 provided data on the relation of NLR to DR incidence (11, 16–18, 26, 28, 30, 32–36, 38–41, 44, 46, 48, 54). Since heterogeneity among studies was substantial (*I²* = 77%, *p* < 0.00001), a random-effects model was leveraged (**Figure 2A**). The results demonstrated a marked relation of rising NLR levels to increased DR incidence, with higher NLR values correlating with greater DR incidence (OR = 1.48, 95% CI: 1.34-1.64; *p* < 0.00001, **Figure 2A**).

#### 3.3.2 NLR and DR incidence (continuous variables)

SMD and 95% CI values were calculated based on extracted mean and SD data of NLR from 49 included studies (11, 16, 17, 24, 25, 27–29, 31–40, 42, 43, 45–54). Due to considerable heterogeneity across eligible articles (*I²=*91%, *p* < 0.00001), the random-effects model was utilized. The pooled SMD estimate showed that a significantly higher value of NLR in DR in comparison to non-DR diabetic cohorts (SMD = 0.47, 95% CI: 0.36-0.59; *p* < 0.00001, **Figure 2B**).

#### 3.3.3 Subgroup analysis

The sources of heterogeneity were explored through subgroup analyses stratified by study design, age, geographical region, and NLR cut-off values. The findings indicated that although NLR showed no significant predictive value for DR incidence in North America (*I²* = 41%, *p* = 0.06), it demonstrated a robust predictive association with DR incidence in all other regions. Subgroup analyses based on study design, age, and NLR cut-off thresholds consistently supported the predictive utility of NLR for DR incidence. Overall, the heterogeneity assessment identified geographical region as the principal contributor to the substantial heterogeneity observed in the pooled DR incidence estimates. Detailed results are presented in **Table 2**.

**Table 2:** Pooled ORs for NLR and DR incidence in subgroup analyses.

Subgroup	DR incidence (categorical variables)			
	Study group	OR [95%CI]	*P* value	*I* ^2^
**Total**	41	1.48 [1.34-1.64]	<0.00001	77%
Study design				
Chort study	5	1.67 [1.24-2.25]	0.0008	72%
Case-control study	36	1.45 [1.31-1.61]	<0.00001	76%
Mean/median age				
≥60y	21	1.30 [1.17-1.44]	<0.00001	68%
<60y	20	1.84 [1.50-2.27]	<0.00001	82%
Region				
Asia	33	1.51 [1.34-1.71]	<0.00001	76%
Europe	5	1.88 [1.57-2.25]	<0.00001	0%
North America	3	1.13 [1.00-1.29]	0.06	41%
NLR cut-off				
≥2.5	10	1.82 [1.35-2.45]	<0.00001	75%
<2.5	20	1.50 [1.27-1.77]	<0.00001	63%

### 3.4 Sensitivity analysis

Result robustness regarding NLR’s clinical significance was rated via sensitivity analysis, which revealed a consistent effect size in the original range following sequential removal of every study. No study exerted disproportionate influence on DR incidence rate (**Supplementary Figures S1A, B**). Therefore, the analysis results were reliable.

### 3.5 Publication bias

**Figure 3:**
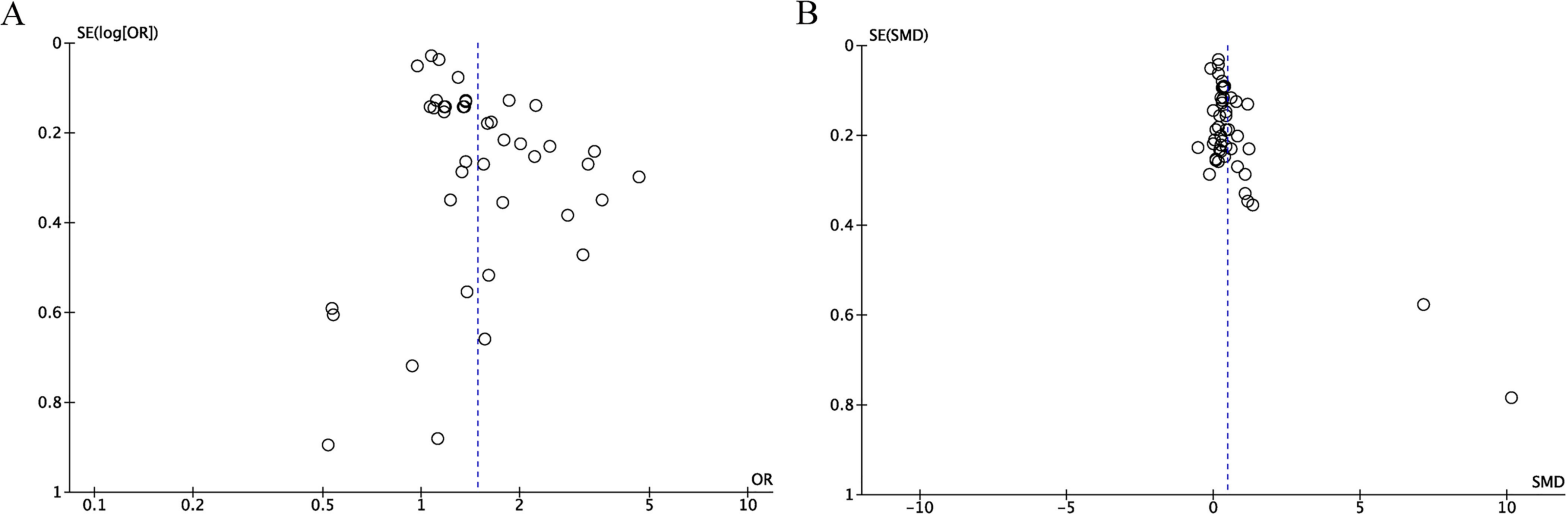
**(A)** Funnel plot for the evaluation of publication bias for DR incidence (categorical variables); **(B)** Funnel plot for the evaluation of publication bias for DR incidence (continuous variables).

Publication bias for the outcome measures was checked via Egger’s test and funnel plots. The publication bias was significant in categorical (Egger: *p* < 0.05) and continuous variables (Egger: *p* < 0.05) of DR incidence. Furthermore, funnel plot asymmetry was observed across these variable types, corroborating the presence of publication bias (**Figures 3A, B**).

## 4 Discussion

Our meta-analysis proved a significant relation of NLR to DR incidence for categorical and continuous variables, indicating a relationship where elevated NLR levels correspond to increased DR risk. Result robustness and reliability were verified via sensitivity and subgroup analyses. However, publication bias might exist, possibly owing to regional variations, population characteristics, and limited sample sizes. Future multinational studies with larger cohorts are warranted to further validate the relation of NLR to DR incidence and mitigate publication bias. In comparison to previous studies by Luo et al. (19) and Harley et al. (20), our study expanded the overall sample size and incorporated the most up-to-date evidence, thereby reaffirming the positive association between NLR and the incidence of DR. By encompassing a broader body of literature, we were able to undertake more comprehensive sensitivity and subgroup analyses, which enabled a more rigorous evaluation of the robustness of our findings and a clearer delineation of the potential sources of heterogeneity.

Hyperglycemia in the T2DM population promotes the expression of transcription factors, leading to neutrophilia and protective lymphocytopenia (55). This hyperglycemia-induced chronic inflammatory response is key in DR development, which arises from the activation of pro-inflammatory pathways regarding polyol accumulation, advanced glycation end products (AGEs), OS, and activated protein kinase C (PKC) (56, 57). These inflammatory pathways upregulate multiple pro-inflammatory factors, like TNF-α, IL-1, vascular endothelial growth factor (VEGF), nuclear factor-kappa B (NF-κB), metabolites of arachidonic acid, adhesion molecules (vascular and cellular), pigment epithelium-derived factor (PEDF), integrins, nitric oxide, as well as components of the complement system. Such mediators activate structures within the retina, including the inner retinal layers, retinal ganglion cells, microglia, and the intraretinal blood barrier (iBRB), along with Müller cells, retinal pigment epithelium (RPE), endothelial cells, as well as pericytes. This activation triggers the recruitment of white blood cells (WBCs) to the vascular endothelium, leading to leukocyte stagnation. Consequently, this process contributes to clinical outcomes like impaired capillary perfusion, detachment of capillaries, vascular permeability, abnormal blood vessel formation, neuronal damage, and DR development (58, 59). In the DR population, persistent inflammation, neurodegeneration, and excessive angiogenesis create a self-perpetuating cycle that exacerbates disease advancement (58).

Wu et al. (60) confirmed the involvement of inflammation in early DR progression by constructing a DR mouse model. They found the presence of inflammatory substances like neutrophils, C-reactive protein (CRP), and interleukin in the mouse’s serum and vitreous fluid, and that inflammatory mediators preceded the formation of neovascularization. Using anti-peroxidase antibodies to immunostain cross-sections of eyes from PDR patients, Binet et al. (61) detected numerous neutrophils near retinal neovascularization, thus confirming neutrophils’ involvement in DR pathology. Another study proves that increased retinal neutrophils in DR damage retinal vascular endothelial cells and disrupt the blood-retinal barrier via a pathway dependent on Fas-Fas ligand (49).

Lessieur et al. (62) proved the relation between neutrophil-derived proteases and early DR development. Neutrophil elastase (NE), a serine protease secreted by neutrophils, shows higher concentrations within the diabetes population (63). Lessieur et al. (62) demonstrated that diabetic mice displayed greater retinal vascular permeability along with increased NE levels in retinal tissue and blood plasma. When NE was genetically removed or selectively blocked in mice with two months of diabetes, it reduced oxidative stress and inflammatory responses in the retina while preventing endothelial cell damage caused by leukocytes. Furthermore, in mice with eight months of diabetes, NE knockout markedly prevented capillary degeneration in the retina induced by diabetic conditions.

In addition, several topical anti-inflammatory medications employed in DR have notable efficacy in slowing disease progression, confirming the strong link of inflammation to DR pathogenesis. Wang et al. (64) examined the effects of intravitreal cyclosporine-A on diabetic retinal differences in diabetic mice and noted that retinal inflammation could be attenuated via inhibition of the pro-inflammatory HMGB-1 protein, which regulates the expression of IL-1β and TNF-α. A study by Rao et al. (65) also demonstrated that local application of a small-molecule inhibitor targeting LFA-1 markedly decreased leukocyte accumulation in the rat retina and prevented blood-retinal barrier breakdown.

Neutrophils are active inflammatory cells that respond nonspecifically. Lymphocytes are protective or regulatory cells. NLR demonstrates the equilibrium between these immune cells, reflecting both immune system activation and broader inflammatory processes. It helps determine whether the body is in uncontrolled inflammation or immune stabilization by quantifying the ratio between two key immune cell types, neutrophils and lymphocytes (12). NLR is closely correlated with traditional inflammatory indicators such as TNF-α, IL-1, and IL-6 (66). Unlike acute inflammatory markers, NLR reflects chronic low-grade inflammation, which represents a long-term pathophysiological hallmark of diabetic complications (67). In a study employing fluorescein angiography, Huang et al. demonstrated that microvascular leakage in patients with diabetic retinopathy was significantly associated with elevated NLR (36). Sustained increases in NLR among individuals with diabetes may therefore contribute to cumulative microvascular injury. These observations suggest that NLR may possess greater clinical utility than acute inflammatory markers for evaluating the gradual progression of diabetic retinopathy over the disease course (68). In contrast to traditional inflammatory factors, NLR is less susceptible to physiological or pathological fluctuations and external variables, exhibits greater stability, and is simpler, faster, and more cost-effective to measure (69). Our study corroborates the significant predictive value of NLR for the incidence of DR, in line with previous research. Notably, our subgroup analyses indicate that geographical region may serve as a key source of heterogeneity.

Diabetes prevalence and its associated complications vary significantly worldwide. The Global Burden of Disease Study noted (70) a global age-standardized diabetes incidence of 6.1% in 2021, with notably higher rates in the North Africa and Middle East super-region (9.3%) and Oceania (12.3%). The incidence of diabetic microvascular complications also varies considerably. The DISCOVER study (2014–2019) (71) reported a global incidence of 18.8% among T2DM individuals, peaking in Europe (23.5%) and dipping to 14.5% in Africa. Furthermore, a 2021 global meta-analysis (4) found high DR rates in Africa (35.90%) and North America/Caribbean (33.30%), contrasting sharply with South and Central America (13.37%). Furthermore, recent evidence suggests potential racial differences in NLR. Ang et al. (72) examined the relationship between demographic characteristics and NLR and reported that, compared with Caucasian individuals, NLR tended to be lower across all other racial groups. Their findings underscore the substantial influence of race on NLR and highlight the importance of considering patient demographic characteristics when applying clinical biomarkers. Such racial variation in NLR may, in turn, contribute to the regional disparities observed in our analyses.

In this study, the incidence of DR (categorical) in North America was not significant. However, no evidence suggests that the results in North America are not statistically valuable because the sample size of North America was the smallest in this study, with only 3 data points. The overall results were strongly influenced by individual studies, possibly causing false positives or false negatives. Our heterogeneity analysis showed that regional differences were the main source of heterogeneity. Subgroup analysis revealed low heterogeneity (*I*^2^ < 50%) in Europe and North America, but high heterogeneity (*I*^2^ > 50%) in other subgroups. This pattern may stem from sample size imbalances: small sample sizes in Europe and America, and significantly larger sample sizes in Asia compared to Europe and America. To address this issue, more international multicenter clinical studies are recommended in the future to reduce the influence of regional selective bias on the conclusions, to verify whether the NLR varies in different regions or in different ethnic groups, and to unravel NLR’s predictive value in DR.

Our study has some limitations. Firstly, all eligible studies were retrospective. Such designs are prone to unmeasured confounders, possibly compromising result accuracy. Moreover, most papers involved small cohorts, raising concerns about selective reporting. Most studies were conducted in Asia, and findings should be cautiously applied to Europe, Africa, the Americas, and other regions. Furthermore, grey literature was excluded, which may introduce publication bias. The large heterogeneity of encompassed studies may also affect the evidence quality.

## 5 Conclusion

Our meta-analysis indicates a possibly significant relationship between NLR and DR incidence, with higher NLR values related to greater DR incidence. Subgroup analyses revealed that the region may affect NLR’s predictive value. Since mostly retrospective studies exhibited large heterogeneity and possible publication bias in this study, international multicenter prospective clinical studies are necessary to corroborate the link of NLR to DR incidence and to explore its optimal suitable population and conditions of use.

## Data Availability

The original contributions presented in the study are included in the article/**Supplementary Material**. Further inquiries can be directed to the corresponding author.

## Supplementary material

Supplementary Table 1 Detailed search strategy in four databases.

Supplementary Table 2 Quality evaluation of the eligible studies with the Newcastle-Ottawa scale.

Supplementary Figure 1 **(A)** Sensitivity analysis of DR incidence (categorical variables); **(B)** Sensitivity analysis of DR incidence (continuous variables).
