# Supplementary material for: Association between the neutrophil-to-lymphocyte ratio and the incidence of diabetic retinopathy: a systematic review and meta-analysis
Source: Front Endocrinol (Lausanne). 2026 Jan 12;16:1712767. doi: 10.3389/fendo.2025.1712767 (PMC12832345; doi:10.3389/fendo.2025.1712767)
Supplement: Supplementary Table 1 — Detailed search strategy in four databases. [file DataSheet1.docx]

**
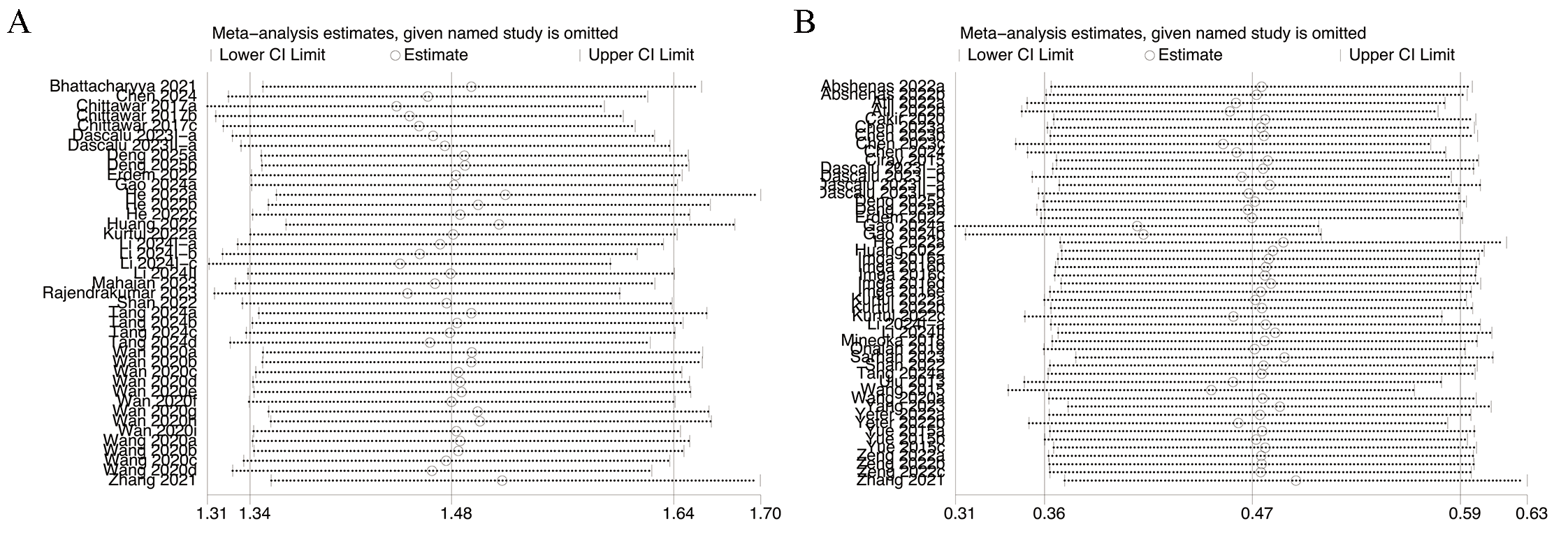
**

**Figure S1** (A) Sensitivity analysis of DR incidence (categorical variables); (B) Sensitivity analysis of DR incidence (continuous variables)

**Table S1** Detailed search strategy in four databases.

| **Database** | **Search Strategy** | **Initial Results** |
| --- | --- | --- |
| PubMed | (((("Neutrophils"[Mesh]) OR (((((((((Neutrophil) OR (Polymorphonuclear Neutrophils)) OR (Polymorphonuclear Neutrophil)) OR (Polymorphonuclear Leukocyte)) OR (Polymorphonuclear Leukocytes)) OR (LE Cells)) OR (LE Cell)) OR (Neutrophil Band Cells)) OR (Neutrophil Band Cell))) AND (("Lymphocytes"[Mesh]) OR (((Lymphocyte) OR (Lymphoid Cells)) OR (Lymphoid Cell)))) AND (Ratio)) AND ((Diabetic Retinopathies) OR ("Diabetic Retinopathy"[Mesh])) | 58 |
| Embase | ((Neutrophils or (Neutrophil or Polymorphonuclear Neutrophils or Polymorphonuclear Neutrophil or Polymorphonuclear Leukocyte or Polymorphonuclear Leukocytes or LE Cells or LE Cell or Neutrophil Band Cells or Neutrophil Band Cell)) and (Lymphocytes or (Lymphocyte or Lymphoid Cells or Lymphoid Cell)) and Ratio and (Diabetic Retinopathies or Diabetic Retinopathy)).af. | 113 |
| Cochrane Library | ((Neutrophils or (Neutrophil or Polymorphonuclear Neutrophils or Polymorphonuclear Neutrophil or Polymorphonuclear Leukocyte or Polymorphonuclear Leukocytes or LE Cells or LE Cell or Neutrophil Band Cells or Neutrophil Band Cell)) and (Lymphocytes or (Lymphocyte or Lymphoid Cells or Lymphoid Cell)) and Ratio and (Diabetic Retinopathies or Diabetic Retinopathy)).af. | 0 |
| Web of Science | ((((Neutrophils) OR (((((((((Neutrophil) OR (Polymorphonuclear Neutrophils)) OR (Polymorphonuclear Neutrophil)) OR (Polymorphonuclear Leukocyte)) OR (Polymorphonuclear Leukocytes)) OR (LE Cells)) OR (LE Cell)) OR (Neutrophil Band Cells)) OR (Neutrophil Band Cell))) AND ((Lymphocytes) OR (((Lymphocyte) OR (Lymphoid Cells)) OR (Lymphoid Cell)))) AND (Ratio)) AND ((Diabetic Retinopathies) OR (Diabetic Retinopathy)) (Topic) | 85 |

**Table S2** Quality evaluation of the eligible studies with Newcastle–Ottawa scale.

| **Study** | **Selection** | | | | **Comparability** | | **Outcome** | | |
| --- | --- | --- | --- | --- | --- | --- | --- | --- | --- |
|  | **Representative-ness** | **Selection of**  **non-exposed** | **Ascertainment**  **of exposure** | **Outcome not present at start** | **Comparability on most important factors** | **Comparability on other risk factors** | **Assessment of outcome** | **Long enough follow-up (median≥1 year)** | **Adequacy**  **(completeness) of follow-up** |
| Tang 2024 | * | * | * | * | * | * | * | * | * |
| Rajendrakumar 2023 | * | * | * | * | * | - | * | * | * |
| *indicates criterion met; - indicates significant of criterion not met. | | | | | | | | | |
| **Study** | **Selection** | | | | **Comparability** | | **Exposure** | | |
|  | **Case definition adequate** | **Representativeness of the cases** | **Selection of controls** | **Definition of controls** | **Comparability on most important factors** | **Comparability on other risk factors** | **Assessment of exposure** | **Same method of ascertainment for cases and controls** | **Non-response rate** |
| Dascalu 2023I | * | * | * | * | - | - | * | * | * |
| Wang 2020 | * | - | * | * | * | - | * | * | * |
| Abshenas 2022 | * | * | * | * | - | - | - | * | * |
| Li 2024I | * | * | * | * | * | - | * | * | * |
| Onalan 2019 | * | - | * | * | - | - | * | * | * |
| Chen 2024 | * | - | * | * | - | - | * | * | * |
| Mahajan 2023 | * | * | * | * | - | - | * | * | * |
| Bhattacharyya 2021 | * | * | * | * | - | - | * | * | * |
| Deng 2025 | * | * | * | * | - | - | * | * | * |
| Wan 2020 | * | * | * | * | * | * | * | * | * |
| Shan 2022 | * | - | * | * | - | - | * | * | * |
| Çakir 2020 | * | - | * | * | - | - | * | * | * |
| Ciray 2015 | * | * | * | * | - | - | * | * | * |
| Mineoka 2018 | * | - | * | * | - | - | * | * | * |
| Chittawar 2017 | * | * | * | * | - | - | * | * | * |
| Wang 2015 | * | * | * | * | - | - | * | * | * |
| Ulu 2013 | * | * | * | * | - | - | * | * | * |
| Zeng 2022 | * | * | * | * | - | - | * | * | * |
| Sarhan 2023 | * | * | * | * | - | - | * | * | * |
| Erdem 2022 | * | * | * | * | - | - | * | * | * |
| Yang 2023 | * | * | * | * | - | - | * | * | * |
| Atli 2022 | * | * | * | * | - | - | * | * | * |
| Li 2024II | * | - | * | * | - | - | * | * | * |
| Zhang 2021 | * | * | * | * | - | - | * | * | * |
| Kurtul 2022 | * | * | * | * | - | - | * | * | * |
| Chen 2023 | * | * | * | * | - | - | * | * | * |
| He 2022 | * | * | * | * | - | * | * | * | * |
| Gao 2024 | * | * | * | * | - | - | * | * | * |
| Yue 2015 | * | * | * | * | - | - | * | * | * |
| Yeter 2022 | * | * | * | * | - | - | * | * | * |
| Dascalu 2023II | * | * | * | * | - | - | * | * | * |
| Huang 2022 | * | * | * | * | - | - | * | * | * |
| İmga 2016 | * | * | * | * | - | - | * | * | * |
| *indicates criterion met; - indicates significant of criterion not met. | | | | | | | | | |
